# Exploring the action of RGDV-gemcitabine on tumor metastasis, tumor growth and possible action pathway

**DOI:** 10.1038/s41598-020-72824-8

**Published:** 2020-09-25

**Authors:** Xiaoyi Zhang, Jinhuan Zhang, Wenchao Liu, Yaonan Wang, Jianhui Wu, Shurui Zhao, Ming Zhao, Shiqi Peng

**Affiliations:** 1 Beijing Area Major Laboratory of Peptide and Small Molecular Drugs, Department of Medicinal Chemistry, School of Pharmaceutical Sciences, Capital Medical University, NO. 10, Youanmenwaixitoutiao, Fengtai District, Beijing, 100069 China; 2 Engineering Research Center of Endogenous Prophylactic of Ministry of Education of China, Department of Medicinal Chemistry, School of Pharmaceutical Sciences, Capital Medical University, Beijing, 100069 China; 3 Beijing Laboratory of Biomedical Materials and Key Laboratory of Biomedical Materials of Natural Macromolecules, Department of Biomaterials, College of Materials Science and Engineering, Beijing University of Chemical Technology, Beijing, 100026 China

**Keywords:** Drug discovery and development, Lead optimization, Drug development

## Abstract

The coupling of Arg-Gly-Asp-Val (RGDV) and gemcitabine led to a hypothesis that the conjugate (RGDV-gemcitabine) could inhibit tumor metastasis. To confirm this hypothesis the activities of RGDV-gemcitabine inhibiting tumor metastasis in vitro and in vivo were presented for the first time. AFM (atomic force microscopy) imaged that RGDV-gemcitabine was able to adhere onto the surface of serum-starved A549 cells, to block the extending of the pseudopodia. Thereby RGDV-gemcitabine was able to inhibit the invasion, migration and adhesion of serum-starved A549 cells in vitro. On C57BL/6 mouse model RGDV-gemcitabine dose dependently inhibited the metastasis of planted tumor towards the lung and the minimal dose was 0.084 µmol/kg/3 days. The decrease of serum TNF-α (tumor necrosis factor), IL-8 (interleukin-8), MMP-2 (matrix metalloprotein-2) and MMP-9 (matrix metalloprotein-9) of the treated C57BL/6 mice was correlated with the action pathway of RGDV-gemcitabine inhibiting the metastasis of the planted tumor towards lung.

## Introduction

Gemcitabine has been one of the clinical first-line drugs for chemotherapy and is a standard choice for treating locally advanced cancer, metastatic pancreatic cancer, breast cancer as well as ovarian cancer^1–6^, however drug resistance, short half-life and side effects seriously decrease its chemotherapeutic efficacy. To enhance the efficacy of treating pancreatic cancer gemcitabine is combined with oxaliplatin, irinotecan, miR-345, nab-paclitaxel, RT11-i anti-body, metformin, ginkgolide B and melatonin^7–13^. To arise the efficacy of treating bladder cancer or muscle invasive bladder cancer gemcitabine is combined with platinum^14,15^. To increase the efficacy of treating advanced breast cancer gemcitabine is combined with carboplatin^16^. To improve the efficacy of treating germ cell cancer, as well as metastatic and unresectable transitional cell carcinoma gemcitabine is combined with sorafenib^17,18^. To heighten the efficacy of treating urothelial carcinoma of the bladder gemcitabine is combined with paclitaxel^19^. To elevate the efficacy of treating concomitant primary lung cancer, metastatic pulmonary colorectal cancer and soft tissue sarcomas gemcitabine is combined with cisplatin, bevacizumab or docetaxel^20,21^. To upgrade the efficacy of treating osteosarcoma, advanced urothelial cancer, metastatic urothelial cancer, bladder cancer, hepatocellular carcinoma and urothelial bladder cancer gemcitabine is combined with licoricidin, taxanes, triptolide, chlorambucil and lentinan^22–26^.

In addition to the combination therapy, previously 4-amino group of gemcitabine was acylated by Arg-Gly-Asp-Val, and 4-(Arg-Gly-Asp-Val-amino)-1-[3,3-difluoro-4-hydroxy-5-hydroxylmethyloxolan-2-yl]pyrimidin-2-one (RGDV-gemcitabine) was evaluated. Comparing to gemcitabine RGDV-gemcitabine had longer half-life, targeted tumor tissues, had no drug resistance, exhibited higher anti-tumor activity, as well as minimally injured kidney, liver and bone marrow. Introducing RGDV was correlated with the advantages of RGDV-gemcitabine over gemcitabine^27^. Based on these issues we hypothesized that RGDV-gemcitabine could be able to inhibit tumor metastasis. To confirm this hypothesis the in vitro and in vivo actions of RGDV-gemcitabine on tumor metastasis and tumor growth were evaluated, and the possible mechanism was discussed.

## Results

### RGDV-gemcitabine does not act on erythrocytes and leucocytes in vitro

**Figure 1:**
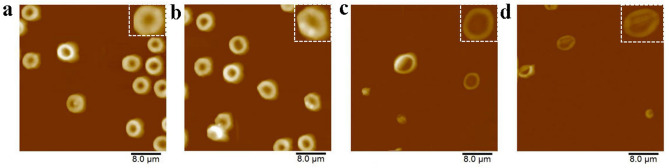
AFM feature of the treated erythrocytes and leucocytes: (**a**) AFM feature of C57BL/6 mouse erythrocytes in ultrapure water of pH 6.7, which is locally amplified and shows a smooth surface; (**b**) AFM feature of C57BL/6 mouse erythrocytes treated with a solution of RGDV-gemcitabine in ultrapure water of pH 6.7 (1 mM), which is locally amplified, shows a smooth surface and suggests that RGDV-gemcitabine does not act on C57BL/6 mouse erythrocytes; (**c**) AFM feature of C57BL/6 mouse leucocytes in ultrapure water of pH 6.7, which is locally amplified and shows a smooth surface; (**d**) AFM feature of C57BL/6 mouse leucocytes treated with a solution of RGDV-gemcitabine in ultrapure water of pH 6.7 (1 mM), which is locally amplified, shows a smooth surface and suggests that RGDV-gemcitabine does not act on C57BL/6 mouse leucocytes.

The AFM images of the erythrocytes (Fig. 1a) and the leucocytes (Fig. 1b) of C57BL/6 mouse in ultrapure water visualized that their surfaces are smooth (see locally amplified erythrocytes and leucocytes also). The AFM images of the erythrocytes (Fig. 1c) and the leucocytes (Fig. 1d) of C57BL/6 mouse in the solution of RGDV-gemcitabine in the ultrapure water (1.0 mM) visualized that their surfaces are also smooth (see locally amplified erythrocytes and leucocytes also). The images suggest that RGDV-gemcitabine does not act on the erythrocytes and the leucocytes of C57BL/6 mouse.

### RGDV-gemcitabine changes surface morphology of A549 cells in vitro

**Figure 2:**
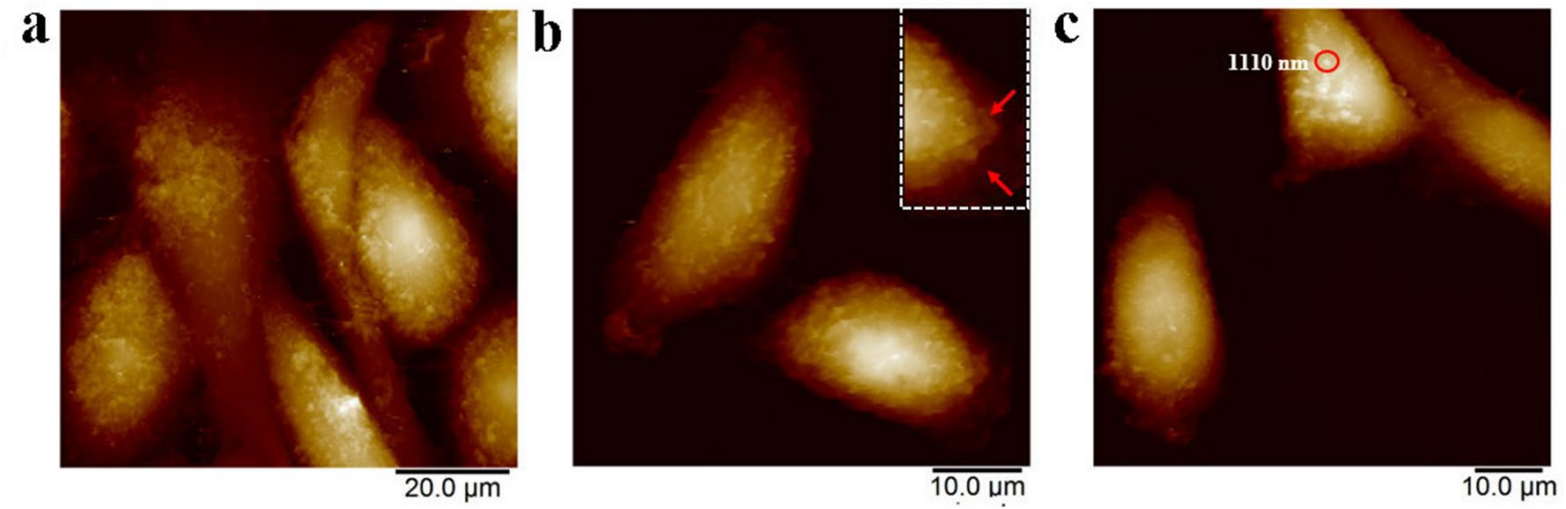
AFM image and the effect of RGDV-gemcitabine on surface morphology of A549 cells: (**a**) AFM image of A549 cells in RPMI-1640 medium with 10% FBS, of which the surfaces are smooth and have no pseudopodia; (**b**) AFM image of A549 cells in RPMI-1640 medium without 10% FBS, the locally amplified illustration shows conical pseudopodia that are highlighted with red arrowhead and suggests that serum starvation A549 cells are able to extend pseudopodia; (**c**) AFM image of 0.5 μM of RGDV-gemcitabine treated A549 cells in serum starvation, of which the surfaces are smooth without pseudopodia and on the surface there are some nano-particles.

To show the effect of RGDV-gemcitabine on the surface morphology of A549 cells the AFM image was recorded. The AFM images of A549 cells in RPMI-1640 medium with 10% FBS (fetal bovine serum) are shown in Fig. 2a, the surfaces of A549 cells are smooth and have no pseudopodium. The AFM images of A549 cells in RPMI-1640 medium without FBS (serum starvation) are shown in Fig. 2b, the surfaces of serum starvation A549 cells are not smooth and have pseudopodium. The AFM images of 0.5 μM of RGDV-gemcitabine treated A549 cells in RPMI-1640 medium without FBS (serum starvation) are shown in Fig. 2c, the surfaces of serum starvation A549 cells are smooth and have no pseudopodium, on which some nano-particles of RGDV-gemcitabine are found. The differentiation images suggest that RGDV-gemcitabine is able to change surface morphology of serum starvation A549 cells, which can be characterized by RGDV-gemcitabine blocking A549 cells to extend pseudopodia and the accumulating nano-particles on the surface of A549 cells.

### RGDV-gemcitabine inhibits the migration of A549 cells in vitro

**Figure 3:**
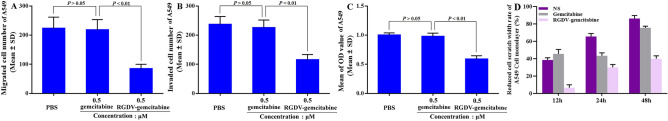
Effect of 0.5 μM of RGDV-gemcitabine on the migration, invasion, adhesion and monolayer of A549 cells: (**a**) effect of 0.5 μM of RGDV-gemcitabine on the migration of A549 cells, which shows that the migration cells induced by 0.5 μM of RGDV-gemcitabine are significantly lower than the migration cells induced by 0.5 μM of gemcitabine and PBS; (**b**) effect of 0.5 μM of RGDV-gemcitabine on the invasion of A549 cells, which shows that the invasion cells induced by 0.5 μM of RGDV-gemcitabine are significantly lower than the invasion cells induced by 0.5 μM of gemcitabine and PBS; (**c**) effect of 0.5 μM of RGDV-gemcitabine on the adhesion of A549 cells, which shows that the adhesion cells induced by 0.5 μM of RGDV-gemcitabine are significantly lower than the adhesion cells induced by 0.5 μM of gemcitabine and PBS; (**d**) effect of 0.5 μM of RGDV-gemcitabine on scratch width of A549 cell monolayer, showing that the potency of 0.5 μM of RGDV-gemcitabine decreasing the scratch width of A549 cell monolayer is significantly lower than those of 0.5 μM of gemcitabine and PBS decreasing the scratch width of A549 cell monolayer ; n = 3.

To show the effect of RGDV-gemcitabine on the migration of A549 cells the chamber system assay was performed. As seen, the migrated number of A549 cells treated with 0.5 μM of gemcitabine is equal to that of A549 cells treated with PBS (phosphate buffer saline), and is significantly higher than that of A549 cells treated with 0.5 μM of RGDV-gemcitabine (Fig. 3a). This means that RGDV-gemcitabine, but not gemcitabine, effectively inhibits the migration of A549 cells.

### RGDV-gemcitabine inhibits the invasion of A549 cells in vitro

To show the effect of RGDV-gemcitabine on the invasion of A549 cells the chamber system assay was performed. As seen, the invasion number of A549 cells treated with 0.5 μM of gemcitabine is equal to that of A549 cells treated with PBS, and is significantly higher than that of A549 cells treated with 0.5 μM of RGDV-gemcitabine (Fig. 3b). This means that RGDV-gemcitabine, but not gemcitabine, effectively inhibits the invasion of A549 cells.

### RGDV-gemcitabine inhibits the adhesion of A549 cells in vitro

To show the effect of RGDV-gemcitabine on the adhesion of A549 cells the chamber system assay was performed. As seen, the adhesion ratio of A549 cells treated with 0.5 μM of gemcitabine is equal to that of A549 cells treated with PBS, and is significantly higher than that of A549 cells treated with 0.5 μM of RGDV-gemcitabine (Fig. 3c). This means that RGDV-gemcitabine, but not gemcitabine, effectively inhibits the adhesion of A549 cells.

### RGDV-gemcitabine changes the monolayer of A549 cells in vitro

To show the effect of RGDV-gemcitabine on the monolayer of A549 cells the wound healing assay was performed. As seen, 12 h, 24 h and 48 h after the incubations the percentage of the reduction in scratch width of A549 cell monolayer treated with 0.5 μM of RGDV-gemcitabine are significantly lower than those of the reduction in scratch width of A549 cell monolayer treated with 0.5 μM of gemcitabine and PBS (Fig. 3d). This means that RGDV-gemcitabine, but not gemcitabine, effectively changes the migration of A549 cell monolayer.

### RGDV-gemcitabine inhibits tumor metastasis in vivo

**Figure 4:**
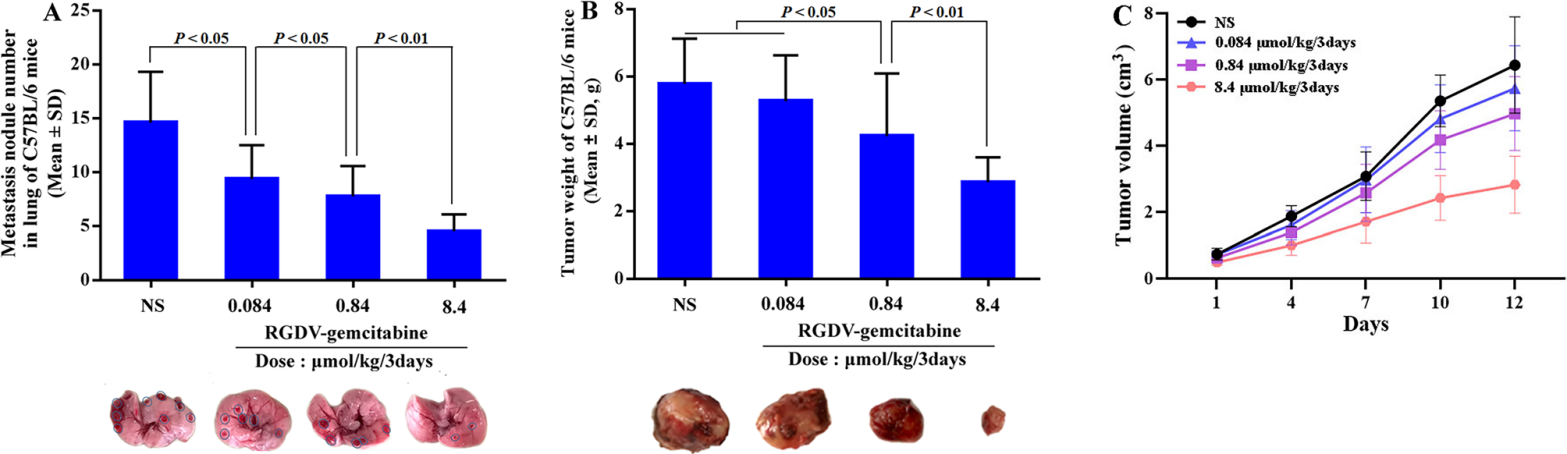
Effect of RGDV-gemcitabine on tumor lung metastasis, tumor weight and tumor volume of LLC sarcoma implanted C57BL/6 mice: (**a**) effect of RGDV-gemcitabine on the metastasis of the tumor towards the lungs of C57BL/6 mice, which is represented with the nodules number occurring on the lungs; (**b**) effect of RGDV-gemcitabine on the weight of the implanted tumor of C57BL/6 mice; (**c**) effect of RGDV-gemcitabine on the volume of the implanted tumor of C57BL/6 mice, which is illustrated with tumor growth curve; n = 12.

The advantages of RGDV-gemcitabine over gemcitabine are not only reflected by the inhibition of the migration, invasion and adhesion of A549 cells in vitro (Fig. 3) but also by the potency of RGDV-gemcitabine in subcutaneous tumors implanted assay of C57BL/6 mouse (12 mice per group). As seen, the number of nodules metastasized to the lungs of the mice treated with RGDV-gemcitabine is significantly less than the number of nodules metastasized to the lungs of C57BL/6 mice treated with NS. For RGDV-gemcitabine the number of nodules metastasized to the lungs is significantly and gradually decreased with the increase of its dose, showing a dose dependent manner and giving a minimal effective dose of 0.084 μmol/kg/3 days (Fig. 4a).

### RGDV-gemcitabine decreases tumor weight in vivo

The in vivo advantage of RGDV-gemcitabine is reflected by the tumor weight of LLC sarcoma implanted C57BL/6 mice (12 mice per group). As seen, the tumor weight of the mice treated with RGDV-gemcitabine is significantly lower than that of the mice treated with NS. For RGDV-gemcitabine the tumor weight is significantly and gradually decreased with the increase of its dose, showing a dose dependent manner and giving a minimal effective dose of 0.84 μmol/kg/3 days (Fig. 4b). It was also found that the tumor weight of the mice treated with gemcitabine (8.4 μmol/kg/3 days, four administrations totally) was equal to that of the mice treated with NS (the dada were not shown here).

### RGDV-gemcitabine decreases tumor volumn in vivo

The in vivo advantage of RGDV-gemcitabine is reflected by tumor volume of LLC sarcoma implanted C57BL/6 mice (12 mice per group). The tumor growth curve of 12 consecutive days of measurements indicates that the tumor volume of the mice treated with RGDV-gemcitabine is significantly lower than that of the mice treated with NS. For RGDV-gemcitabine the tumor volume is significantly and gradually decreased with the increase of its dose, showing a dose dependent manner and giving a minimal effective dose of 0.84 μmol/kg/3 days (Fig. 4c). It was also found that the tumor volume of the mice treated with gemcitabine (8.4 μmol/kg/3 days, four administrations totally) was equal to that of the mice treated with NS (the dada were not shown here).

### RGDV-gemcitabine decreases serum MMP-2 and MMP-9 in vivo

**Figure 5:**
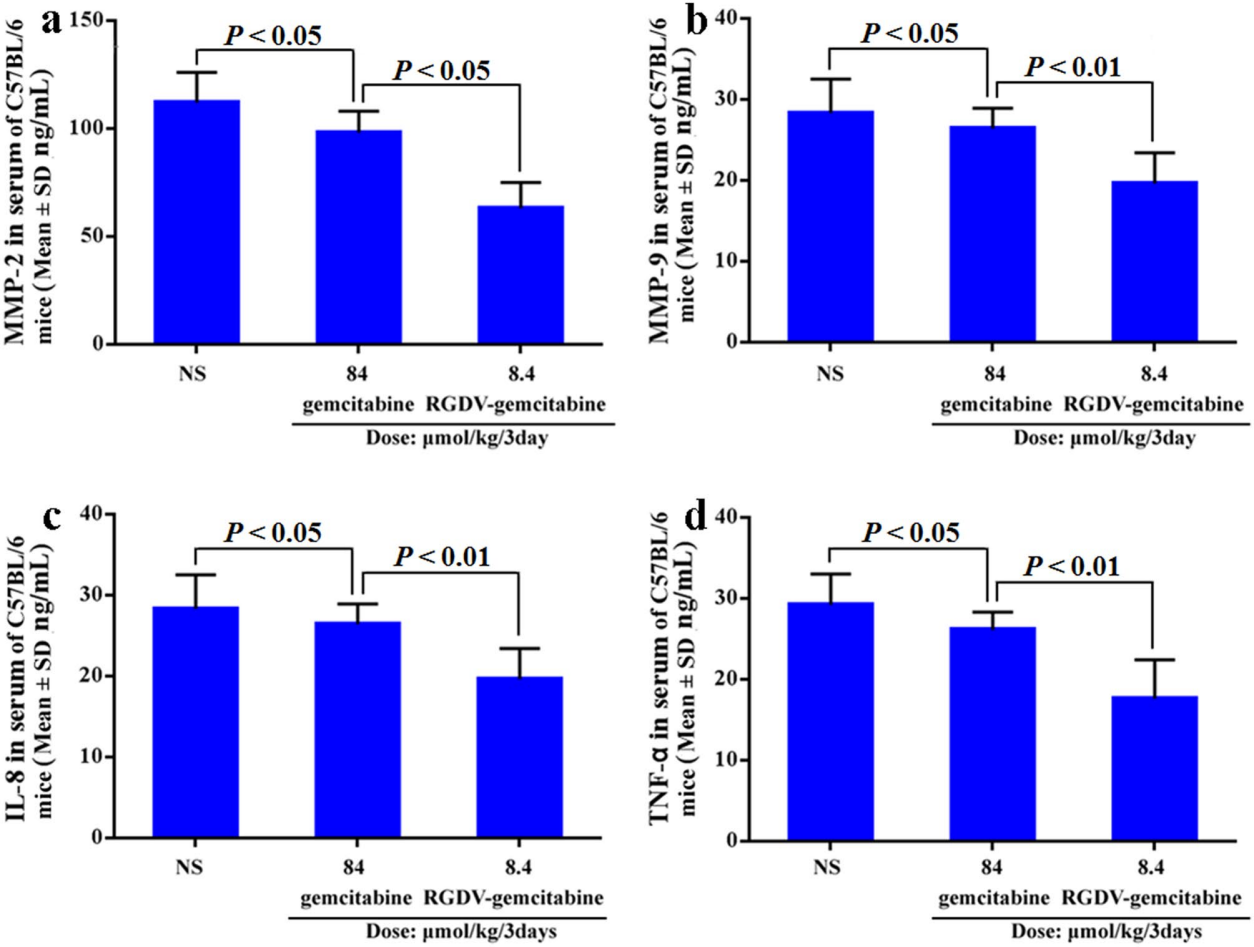
Levels of MMP-2, MMP-9, IL-8 and TNF-α in the serum of C57BL/6 mice orally treated by 8.4 μmol/kg/3 days (four administrations totally) of RGDV-gemcitabine: (**a**) levels of MMP-2 in the serum of C57BL/6 mice orally treated by RGDV-gemcitabine, which shows that the activity of RGDV-gemcitabine is more than 10 times of that of gemcitabine; (**b**) levels of MMP-9 in the serum of C57BL/6 mice orally treated by RGDV-gemcitabine, which shows that the activity of RGDV-gemcitabine is more than 10 times of that of gemcitabine; (**c**) levels of IL-8 in the serum of C57BL/6 mice orally treated by RGDV-gemcitabine, which shows that the activity of RGDV-gemcitabine is more than 10 times of that of gemcitabine; (**d**) levels of TNF-α in the serum of C57BL/6 mice orally treated by RGDV-gemcitabine, which shows that the activity of RGDV-gemcitabine is more than 10 times of that of gemcitabine; n = 12.

The characteristics of RGDV-gemcitabine and gemcitabine led to the measurement of the levels of MMP-2 and MMP-9 in the serum of C57BL/6 mice treated with RGDV-gemcitabine (8.4 μmol/kg/3 days) and gemcitabine (84 μmol/kg/3 days), four administrations totally. As seen, the level of MMP-2 of the mice treated with gemcitabine is significantly lower than that of the mice treated with NS and is significantly higher than that of the mice treated with RGDV-gemcitabine (Fig. 5a). Similarly, the level of MMP-9 of the mice treated with gemcitabine is significantly lower than that of the mice treated with NS and is significantly higher than that of the mice treated with RGDV-gemcitabine (Fig. 5b). However, the levels of MMP-2 and MMP-9 in the serum of the mice treated with NS are equal to those of the mice treated with 8.4 μmol/kg/3 days of gemcitabine (the dada were not shown here). Thus at the doses of inhibiting tumor metastasis RGDV-gemcitabine, but not gemcitabine, effectively decrease of serum MMP-2 and MMP-9 of C57BL/6 mice.

### RGDV-gemcitabine decreases serum IL-8 and TNF-ɑ in vivo

The characteristics of RGDV-gemcitabine and gemcitabine led to the measurement of the levels of IL-8 and TNF-ɑin the serum of C57BL/6 mice treated with RGDV-gemcitabine (8.4 μmol/kg/3 days) and gemcitabine (84 μmol/kg/3 days), four administrations totally. As seen, the level of IL-8 of the mice treated with gemcitabine is significantly lower than that of the mice treated with NS and is significantly higher than that of the mice treated with RGDV-gemcitabine (Fig. 5c). Similarly, the level of TNF-ɑ of the mice treated with gemcitabine is significantly lower than that of the mice treated with NS and is significantly higher than that of the mice treated with RGDV-gemcitabine (Fig. 5d). However, the levels of IL-8 and TNF-ɑ in the serum of the mice treated with NS are equal to those of the mice treated with 8.4 μmol/kg/3 days of gemcitabine (the dada were not shown here). Thus at the doses of inhibiting implanted tumor growth RGDV-gemcitabine, but not gemcitabine, effectively decrease of serum IL-8 and TNF-ɑ of C57BL/6 mice.

## Discussion

**Figure 6:**
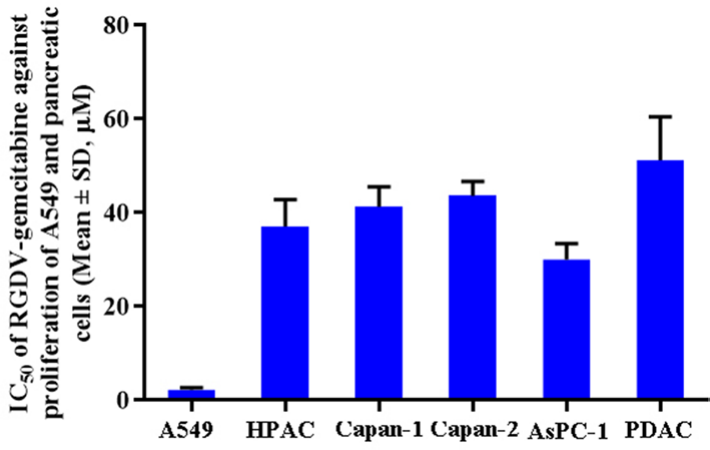
IC_50_ of RGDV-gemcitabine against the proliferation of A549 and pancreatic tumor cells, which shows that A549 is the most sensitive one to RGDV-gemcitabine, n = 9.

In order to select cells associated with tumor metastasis we conducted the preliminary experiment. In the preliminary experiment adenocarcinoma human alveolar basal epithelial cells (A549), human pancreatic tumor cell lines (AsPC-1, Capan-1 and Capan-2), human acinar epithelial carcinoma cell (HPAC) and pancreatic ductal adenocarcinoma cell (PDAC) were involved. We found that among the cancer cell lines A549 was the most sensitive one to RGDV-gemcitabine (see Fig. 6). In this case A549 cells were used for in vitro assay.

We showed that 0.5 μM of RGDV-gemcitabine, but not 0.5 μM of gemcitabine, effectively inhibited the migration, invasion and adhesion of A549 cells in vitro^28^. We also showed that 0.5 μM of RGDV-gemcitabine, but not 0.5 μM of gemcitabine, effectively inhibited the wound healing of the monolayer of A549 cells in vitro. It is generally accepted that the migration, invasion, adhesion and monolayer wound healing of A549 cells depend on pseudopodia extension. Thus we suggested that if RGDV-gemcitabine inhibiting migration, invasion, adhesion and the monolayer wound healing of serum starvation A549 cells can be thought of as event stream, then the nano-particles of RGDV-gemcitabine adhering on the surfaces and blocking pseudopodia extension of serum starvation A549 cells should be triggering events or source events. Since RGDV-gemcitabine did not change the morphology of the erythrocytes and leucocytes, the action of RGDV-gemcitabine on serum starvation A549 cells reflected a selectivity of its nano-particles to serum starvation A549 cells.

As a result of this selective adhesion the in vivo advantages of RGDV-gemcitabine over gemcitabine was logically revealed. In vivo RGDV-gemcitabine, but not gemcitabine, dose dependently inhibited the metastasis of the implanted tumor towards the lung of C57BL/6 mice, slowed the increase of the weight and volume of implanted tumor of C57BL/6 mice.

It is generally accepted that MMP-2 and MMP-9 are involved in tumor metastasis. This profile led to the measurement of serum MMP-2 and MMP-9 of C57BL/6 mice. We showed that 8.4 μmol/kg/3 days of RGDV-gemcitabine, but not 8.4 μmol/kg/3 days of gemcitabine, decreased the levels of MMP-2 and MMP-9 in the serum of C57BL/6 mice. Therefore the down regulation of serum MMP-2 and MMP-9 may be a possible action pathway of RGDV-gemcitabine inhibiting tumor metastasis.

It is generally accepted that IL-8 and TNF-ɑ are involved in tumor growth. This profile led to the measurement of serum IL-8 and TNF-ɑ of C57BL/6 mice. We showed that 8.4 μmol/kg/3 days of RGDV-gemcitabine, but not 8.4 μmol/kg/3 days of gemcitabine, decreased the levels of IL-8 and TNF-ɑ in the serum of C57BL/6 mice. Therefore the down regulation of serum IL-8 and TNF-ɑ may be a possible action pathway of RGDV-gemcitabine inhibiting the growth of the implanted tumor.

## Conclusion

In conclusion, the accumulation of RGDV-gemcitabine and the lack of the extending pseudopodia on the surface of the serum-starved A549 cells lead to dual actions of RGDV-gemcitabine inhibiting the metastasis of the implanted tumor towards the lung of the treated C57BL/6 mice and slowing the growth of the implanted tumor in the treated C57BL/6 mice. Lung cancer is one of the mostly diagnosed cancers, the dual action of RGDV-gemcitabine inhibiting tumor metastasis and slowing tumor growth is of clinical importance.

## Materials and methods

### Reagents and instruments

For this work gemcitabine RGDV-gemcitabine was prepared by following the literature^27^. A 99.5% purity of HPLC (high-performance liquid chromatography) of RGDV-gemcitabine was conducted on an Agilent Technologies 1200 Series HPLC system (Agilent Technologies, Santa Clara, CA, USA) by using Eclipse XDB C18 column (5 μm, 4.6 mm × 150 mm, 40 °C), and was eluted with aqueous methanol in a gradient consisting of 60% methanol (0–5 min), 70% methanol (5–10 min), 80% methanol (10–20 min) and 90% methanol (20–30 min). A 0.8 mL/min flow rate was used.

### Animals, cells and ethics

LLC and A549 cells were purchased from keyGEN BioTECH (Nanjing China). From the Laboratory Animal Center of Capital Medical University male C57BL/6 mice (22 ± 2 g) were purchased. Ethics Committee of Capital Medical University assured that C57BL/6 mouse welfare filled the requirements of the Animal Welfare Act and NIH Guide for Care and Use of Laboratory Animals. All in vitro assay on A549 cells and in vivo assay on C57BL/6 mice were based on the protocols reviewed and approved by the committee. Statistical analyses of all data were carried out by use of one-way ANOVA (analysis of variance). All analyses were done with SPSS 19.0 program and *P* value < 0.05 was considered statistically significant.

### A549 cell monolayer assay

The effect of RGDV-gemcitabine on A549 cell monolayer was assayed with wound healing model^29^. In brief, in a six-well plate A549 cells were at 37 °C and in 5% CO_2_ grown for 24 h to reach a confluence of 80–90%. Then the cells were seeded into a 6-well tissue culture plate and in the presence of 0.1% DMSO (dimethyl sulfoxide) or gemcitabine (0.5 μM) or RGDV-gemcitabine (0.5 μM) were incubated for 0 h, 12 h, 24 h and 48 h to form a cell monolayer. At the time points of 0 h, 12 h, 24 h and 48 h incubations the scratch across cell monolayer were made by using a sterile 10 μL pipette tip, and the wound gap was photographed to get scratch width and calculate the relative ration of width reduction.

### Cell migration assay

The effect of RGDV-gemcitabine on cancer cell migration was evaluated with cancer cell migration assay^30^. In brief, 100 μL of RPMI-1640 medium containing A549 cells (4 × 10^5^ cells/mL) were added into the top chamber, while 600 μL of RPMI-1640 medium supplemented with 10% FBS were placed in the bottom chamber. At 37 °C the wells in the top chamber were treated with DMSO (0.1%, blank control) or gemcitabine (0.5 μM, positive control) or RGDV-gemcitabine (0.5 μM) for 8 h. The remained matrigel and cells in the top chamber were removed by cotton swabs. Then A549 cells on the bottom surface of the membrane were fixed with paraformaldehyde for 30 min and stained with 0.5% crystal violet for 15 min. The migrated cells on the membrane were washed with ultrapure water and photographed under an optical microscope. The cells were counted in at least 9 random microscopic fields. Each assay was repeated for three times.

### Cell invasion assay

This assay was done by using a chamber system based procedure of the literature^31^. In brief, at 37 °C matrigel matrix (BD Biosciences) was placed into the transwell filter (100 μL/well, 8.0 μm PET, Millipore) and allowed to complete 1-h-gelation. Into the wells of the top chamber 150 μL of RPMI-1640 medium containing A549 cells (4 × 10^5^ cells /mL) were added, and into the bottom chamber 600 μL of RPMI-1640 medium supplemented with 10% FBS were added. At 37 °C the wells in the top chamber were treated with DMSO (0.1%, blank control) or gemcitabine (0.5 μM, positive control) or RGDV-gemcitabine (0.5 μM) for 12 h. The remained matrigel and cells in the top chamber were removed by cotton swabs, while A549 cells on the bottom surface of the membrane were fixed with paraformaldehyde for 30 min and stained with 0.5% crystal violet for 15 min. The invading cells on the membrane were washed with ultrapure water and photographed under an optical microscope. The cells were counted in at least 9 random microscopic fields. Each assay was repeated three times.

### Cell adhesion assay

This assay was performed by using FN (fibronectin) (10 μg/mL) coated 96-well plate and by use the procedure of literature^32^. In brief, the suspension of A549 cells in RPMI-1640 medium supplemented with 10% FBS (100 μL, 5 × 10^4^ cells/mL) was added into FN coated 96-well plate, and into each well 25 μL of PBS or a solution of gemcitabine in PBS (0.5 μM, 25 μL, positive control) or a solution of RGDV-gemcitabine in PBS (0.5 μM, 25 μL) were added to incubate for 1 h. Finally the non-adherent cells were washed by PBS, the value of OD (optical density) of the well was tested with MTT (3-(4,5-dimethylthiazol-2-yl)-2,5-diphenyltetrazolium bromide) method and the adhesive ratio of A549 cells was calculated.

### In vivo LLC sarcoma metastasis assay

This assay was carried out with 8-week-old male C57BL/6 mice by using a procedure of the literature^33,34^. In brief, 2 × 10^7^ viable LLC cells were suspended in 0.2 mL of NS, this suspension was injected into the skin of the right armpit of C57BL/6 mice to form subcutaneous solid tumors. Nine days after injection the tumor volume reached ~ 50 mm^3^ and the mice were randomly divided to NS group (oral dose: 10 mL/kg/3 days, totally 4 administrations) and RGDV-gemcitabine group (oral dose: 8.4, 0.84, and 0.084 μmol/kg/3 days, four administrations totally), during which the tumor volume was measured with a caliper every 3 days. Forty eight hours after the last administration, all C57BL/6 mice were weighed. After ether anesthesia the mice were sampled blood, killed and immediately dissected to obtain the tumor tissues and lungs. The tumor tissue was weighed to represent the anti-tumor activity. The lung was used to count the metastasis nodule.

### Serum MMP-2, MMP-9, IL-8 and TNF-α assays

Serum MMP-2, MMP-9, IL-8 and TNF-α of the C57BL/6 mice orally treated with RGDV-gemcitabine (8.4 μmol/kg/3 days, four administrations totally) were measured by following the guidance of mouse total MMP-2 ELISA kit (R&D Systems, Inc., USA), mouse total MMP-9 ELISA kit (R&D Systems, Inc., USA), mouse IL-8 ELISA kit (Xitang Biotechnology Co., Shanghai, People’s Republic of China) and mouse TNF-α ELISA kit (Xitang Biotechnology Co., Shanghai, People’s Republic of China) respectively.

## References

[1] Adamska A (2017). Molecular and cellular mechanisms of chemoresistance in pancreatic cancer. Adv. Biol. Regul..

[2] Ma JL, Hui PP, Meng WY, Wang N, Xiang SH (2017). Ku70 inhibits gemcitabine-induced DNA damage and pancreatic cancer cell apoptosis. Biochem. Biophys. Res. Commun..

[3] Cavalcante LS, Monteiro G (2014). Gemcitabine: metabolism and molecular mechanisms of action, sensitivity and chemoresistance in pancreatic cancer. Eur. J. Pharmacol..

[4] Garcia-Cremades M, Pitou C, Iversen P, Troconiz I (2018). Predicting tumour growth and its impact on survival in gemcitabine-treated patients with advanced pancreatic cancer. Eur. J. Pharm. Sci..

[5] Dang C (2017). MUC-king with HIF may rewire pyrimidine biosynthesis and curb gemcitabine resistance in pancreatic cancer. Cancer Cell.

[6] Jia YF, Xie JW (2015). Promising molecular mechanisms responsible for gemcitabine resistance in cancer. Genes Dis..

[7] Gruenberg J, Manivel C, Gupta P, Dykoski R, Mesa H (2016). Fatal acute cardiac vasculopathy during cisplatin–gemcitabine–bevacizumab (CGB) chemotherapy for advanced urothelial carcinoma. J. Infect. Chemother..

[8] Uz M (2019). Dual delivery nanoscale device for miR-345 and gemcitabine co-delivery to treat pancreatic cancer. J. Control Release.

[9] Rivera F (2018). Tumor treating fields in combination with gemcitabine or gemcitabine plus nab-paclitaxel in pancreatic cancer: results of the PANOVA phase 2 study. Pancreatology.

[10] Kang YW (2018). KRAS targeting antibody synergizes anti-cancer activity of gemcitabine against pancreatic cancer. Cancer Lett..

[11] Qian WK (2018). Metformin suppresses tumor angiogenesis and enhances the chemosensitivity of gemcitabine in a genetically engineered mouse model of pancreatic cancer. Life Sci..

[12] Lou CJ, Lu HB, Ma ZG, Liu C, Zhang YQ (2019). Ginkgolide B enhances gemcitabine sensitivity in pancreatic cancer cell lines via inhibiting PAFR/NF-кB pathway. Biomed. Pharmacother..

[13] Leja-Szpak A (2018). Melatonin and its metabolite N1-acetyl-N2-formyl-5-methoxykynuramine (afmk) enhance chemosensitivity to gemcitabine in pancreatic carcinoma cells (PANC-1). Pharmacol. Rep..

[14] Parikh M (2018). Pembrolizumab combined with either docetaxel or gemcitabine in patients with advanced or metastatic platinum-refractory urothelial cancer: results from a phase I study. Clin. Genitourin. Cancer..

[15] Goel S (2019). Role of gemcitabine and cisplatin as neoadjuvant chemotherapy in muscle invasive bladder cancer: experience over the last decade. Asian J. Urol..

[16] Vernieri C (2019). Single-agent gemcitabine vs carboplatin–gemcitabine in advanced breast cancer: a retrospective comparison of efficacy and safety profiles. Clin. Breast Cancer.

[17] Hurwitz M (2018). Multicenter phase 2 trial of gemcitabine, carboplatin, and sorafenib in patients with metastatic or unresectable transitional-cell carcinoma. Clin. Genitourin. Cancer.

[18] McKenzie H (2018). Salvage chemotherapy with gemcitabine, paclitaxel, ifosfamide, and cisplatin for relapsed germ cell cancer. Clin. Genitourin. Cancer.

[19] Fukuhara H (2017). Successful treatment with paclitaxel, carboplatin, and gemcitabine as second-line chemotherapy for recurrent urothelial carcinoma of the bladder with glandular differentiation after radical cystectomy: a case report. Urol. Case Rep..

[20] Chen HM (2015). Concomitant primary lung cancer and metastatic pulmonary colorectal cancer that responded to gemcitabine/cisplatin/bevacizumab combination therapy. J. Cancer Res. Pract..

[21] Ducoulombier A, Cousin S, Kotecki N, Penel N (2016). Gemcitabine-based chemotherapy in sarcomas: a systematic review of published trials. Crit. Rev. Oncol. Hematol..

[22] Wang YF, Wang SL, Liu JH, Lu YX, Li DH (2018). Licoricidin enhances gemcitabine-induced cytotoxicity in osteosarcoma cells by suppressing the Akt and NF-κB signal pathways. Chem. Biol. Interact..

[23] Giannatempo P (2016). The impact of adding taxanes to gemcitabine and platinum chemotherapy for the first-line therapy of advanced or metastatic urothelial cancer: a systematic review and meta-analysis. Eur. Urol..

[24] Yang Y (2018). Synergistic antitumour effects of triptolide plus gemcitabine in bladder cancer. Biomed. Pharmacother..

[25] Fan ML (2015). Chlorambucil gemcitabine conjugate nanomedicine for cancer therapy. Eur. J. Pharm. Sci..

[26] Sun M, Zhao WY, Xie QP, Zhan YH, Wu B (2015). Lentinan reduces tumor progression by enhancing gemcitabine chemotherapy in urothelial bladder cancer. Surg. Oncol..

[27] Liu W (2019). RGDV-modified gemcitabine: a nano-medicine capable of prolonging half-life, overcoming resistance and eliminating bone marrow toxicity of gemcitabine. Int. J. Nanomed..

[28] Wang ZM (2018). Gemcitabine-loaded gold nanospheres mediated by albumin for enhanced anti-tumor activity combining with CT imaging. Mater. Sci. Eng. C.

[29] Yalcin TE, Ilbasmis-Tamer S, Takka S (2018). Development and characterization of gemcitabine hydrochloride loaded lipid polymer hybrid nanoparticles (LPHNs) using central composite design. Int. J. Pharm..

[30] Borsoi C (2017). Gemcitabine enhances the transport of nanovector-albumin-bound paclitaxel in gemcitabine-resistant pancreatic ductal adenocarcinoma. Cancer Lett..

[31] Vale N (2017). Gemcitabine anti-proliferative activity significantly enhanced upon conjugation with cell-penetrating peptides. Bioorg. Med. Chem. Lett..

[32] Birhanu G, Javar HA, Seyedjafari E, Zandi-Karimi A (2017). Nanotechnology for delivery of gemcitabine to treat pancreatic cancer. Biomed. Pharmacother..

[33] Zhou Y (2018). Sensitive analysis and pharmacokinetic study of a novel gemcitabine carbamate prodrug and its active metabolite gemcitabine in rats using LC–ESI–MS/MS. J. Chromatogr. B.

[34] Dubey RD, Saneja A, Gupta P, Gupta P (2016). Recent advances in drug delivery strategies for improved therapeutic efficacy of gemcitabine. Eur. J. Pharm. Sci..

